# Employment trajectories until midlife in schizophrenia and other psychoses: the Northern Finland Birth Cohort 1966

**DOI:** 10.1007/s00127-022-02327-6

**Published:** 2022-07-07

**Authors:** Tuomas Majuri, Anni-Emilia Alakokkare, Marianne Haapea, Tanja Nordström, Jouko Miettunen, Erika Jääskeläinen, Leena Ala-Mursula

**Affiliations:** 1 Center for Life Course Health Research, University of Oulu, P.O. Box 5000, 90014 Oulu, Finland; 2 Medical Research Center Oulu, Oulu University Hospital and University of Oulu, Oulu, Finland; 3 Department of Psychiatry, Oulu University Hospital, Oulu, Finland; 4 Northern Finland Birth Cohorts, Infrastructure for Population Studies, University of Oulu, Oulu, Finland

**Keywords:** Schizophrenia, Psychosis, Employment, Trajectory, Follow-up, Outcome

## Abstract

**Purpose:**

Psychoses are associated with poor labour market attachment, but few studies have compared schizophrenia (SZ) and other psychoses (OP). Moreover, studies on long-term employment trajectories over individuals’ working life courses are lacking. We compared 30 year employment trajectory patterns in a general population sample among individuals with SZ, OP, and those with no psychosis (NP).

**Methods:**

Utilising the Northern Finland Birth Cohort 1966, we collected survey data on employment from ages 16 to 45 and detected individuals with register-based history of SZ (*n* = 62), OP (*n* = 87), or NP (*n* = 6464) until age 46. Through gender-specific latent class analyses on annual employment roles, we identified traditional, highly educated, self-employed, delayed and floundering employment trajectories with distinct socioeconomic characteristics. We addressed attrition by conducting weighted analyses.

**Results:**

Floundering trajectories were common among individuals with SZ (79% of men, 73% of women) and OP (52% of men, 51% of women). In NP, a traditional employee trajectory was most common in men (31%), and a highly educated trajectory in women (28%). A history of psychosis was associated with heightened odds ratios (ORs; 95% confidence intervals (CIs)) for floundering trajectories in both men (SZ: 32.9 (13.3–81.4); OP: 7.4 (4.0–13.9)) and women (SZ: 9.9 (4.6–21.5); OP: 3.9 (2.1–7.1)) compared to NP. Weighted analyses produced similar results.

**Conclusion:**

Most individuals with SZ or OP have floundering employee trajectories reflecting an elevated risk of unemployment and part-time work until midlife. These results indicate the importance of improving labour market attachment during the early phases of psychoses.

**Supplementary Information:**

The online version contains supplementary material available at 10.1007/s00127-022-02327-6.

## Introduction

Psychotic disorders are known to affect individuals already at a young age, often leading to poor long-term attachment to working life [1]. People with schizophrenia (SZ) are at a particularly high risk of being outside the labour market [2], as reflected by their employment rates of 10–30% [3, 4], unemployment rates of 89–95% [5–7] and disability pension rates of 80–89% [6, 7]. In Finland, 50% of SZ patients are granted a disability pension within five years of illness onset [8]. Studies comparing employment in individuals with SZ and those with other psychoses (OP) are rare. Compared to persons with SZ, persons with other non-affective psychoses are somewhat less likely to be unemployed [5, 7] and to receive disability pensions [7].

However, studies on occupational outcomes in psychoses tend to be cross-sectional or cover only a part of working life. Studies on long-term patterns of occupational functioning in SZ are limited, and no studies have specifically focused on employment [9]—that is, how people with SZ develop their careers and adapt to the labour market [10]. Importantly, few studies have concentrated on population-level career development patterns during the work-life course until middle age in relation to psychoses. Examining this information would be useful in raising awareness and developing interventions to improve the long-term occupational outcomes of individuals with psychoses.

To our knowledge, only one previous study [9] investigated the longitudinal employment patterns of people with psychoses. The study, in which 10 year employment trajectories were used, revealed an overall long-term benefit of early intervention services on the employment rate of individuals with schizophrenia-spectrum disorders [9]. The authors found a significantly larger proportion of patients in the good-employment cluster among those who had received early intervention services (68%) than among those who had received standard care (52%) [9]. However, the study adopted a relatively broad definition of employment and did not account for gender differences.

In this study, we aimed to compare the distribution of typical employment trajectories in a general population birth cohort between men and women with SZ, OP and no history of psychosis (NP) until midlife. We stratified our analysis by gender because women and men work in different occupations with varying employment opportunities and because gender may be associated with psychoses’ outcomes [11]. We considered psychosis onset age and a wide range of socioeconomic factors as potential confounders. Influencing individual lives before employment, privileged backgrounds are associated with favourable patterns in later careers [12]. In SZ, strong school performance has been found to be a predictor of the non-receipt of disability pensions [13] and even returning to the labour market after receiving disability pensions [14]. Altogether, using longitudinal survey data on 30 years of working life and register-based diagnosis data, we specifically aimed to estimate the risks among individuals with psychoses of experiencing unfavourable employment trajectories characterised by poorer labour market attachment.

## Methods

### Sample

The study was based on the Northern Finland Birth Cohort 1966 (NFBC1966) [15], which is an unselected, general population sample comprising 12,058 live-born children with expected dates of birth in 1966 in the provinces of Oulu and Lapland. The cohort members were followed up with data collection, including linkages with national register data (see Online supplement 1), at different ages.

The 46 year follow-up survey was conducted in 2012, targeting 10,331 cohort members (86% of the original sample) alive and living in Finland at known addresses. Altogether 6613 individuals (64% of the target population) responded to the questionnaire on work, economy and resources, including annual employment roles between ages 16 and 45, and allowed their data to be used in this research.

### Detecting individuals with a history of psychosis

Psychiatric diagnoses of NFBC1966 members until the 46year follow-up were retrieved from multiple national registers: the Care Register for Health Care (CRHC), the Register of Primary Health Care Visits (2011–), the Social Insurance Institution of Finland (SII), and the Finnish Centre for Pensions (FCP) (1974–) [16]. See Online supplement 1 for further details. The register data were complemented by self-reported lifetime psychosis diagnosis, obtained by asking the participants in the 31- and 46 year NFBC1966 questionnaires whether they had ever been diagnosed by a physician as having psychosis. This yielded additional 18 cases, presumably with mild psychoses, as no hospitalisations in the registered data. These people were assumed to have psychoses other than SZ.

The diagnostic categories based on different versions of the International Classification of Diseases and used in the study were SZ (ICD-8: 2950–2959, 297; ICD-9: 2950–2959, 297; ICD-10: F20, F22, F24, F25) and OP (ICD-8: 2960–2969, 2980–2983, 2988, 2989, 299; ICD-9: 2961E, 2962E, 2963E, 2964E, 2967, 2988, 2989; ICD-10: F23, F28, F29, F302, F312, F315, F323, F333). The focus was on individuals with SZ and OP. For comparison purposes, the remaining cohort members (i.e., persons with NP in the national registers) were used as controls. When setting the diagnosis for each subject, we used a hierarchical system in which the life-time diagnosis was the disorder that had the highest position in the hierarchy, based on severity (Online supplement 1). This hierarchy has been used in previous studies of NFBC1966 [14].

We identified 62 subjects with SZ, 87 subjects with OP and 6,464 subjects with NP who had available employment trajectory information, comprising the final sample (*n* = 6613).

### Measures

#### Annual employment-related roles

To enable latent class analysis of longitudinal employment trajectories, we utilised a working-life-focused life history calendar (LHC) as a part of the 46 year follow-up survey in 2012 [12]. For each year from 1982 to 2011 (ages 16–45), the participants marked whether they had occupied one or more of the following roles: (1) student, (2) full-time employed, (3) part-time employed, (4) self-employed, (5) unemployed, (6) on parental leave or (7) on sabbatical leave or otherwise not working. The LHC survey responses were proven reliable by comparing them to national register employment data [12]. The sample size in the present latent class analysis differed from the original employment trajectory analysis [12] because we used participants’ most recent consents to use their personal data and included updated LHC survey data.

#### Sociodemographic, work-related, and illness-related factors

To describe the respondents’ childhood family’s socioeconomic situation, we used their *fathers’ socio-economic status* (SES), which was classified as either white collar or not, from the 14 year NFBC1966 follow-up.

We gathered data on the participants’ *average school grades* (range 4–10) when leaving compulsory education at the age of 16 years from the register of the Finnish national application system for upper secondary education.

*Educational level, (*i.e., the highest attained educational level by the age of 46 based on the questionnaire) was classified using the International Standard Classification of Education [17]. The basic or below level included early childhood education, primary education and lower secondary education. The secondary level included upper secondary education and post-secondary non-tertiary education. Tertiary education included short-cycle tertiary education, bachelor’s degree or equivalent, master’s degree or equivalent and doctoral degree or equivalent level. *Marital status* at age 46 was requested and dichotomised as: (1) single, divorced, separated or widowed and (2) married, registered or cohabiting. *Socioeconomic status* in 2012 at the age of 46 years was retrieved from the register of Statistics Finland in terms of the following categories: (1) farmers, (2) entrepreneurs, (3) upper white collar, (4) lower white collar, (5) manual workers, (6) students, (7) pensioners and (8) others, mostly unemployed [18].

*Illness onset age,* meaning the age of the first occurrence of psychosis for the SZ and OP groups, was defined by using the CRHC, the register of the FCP, the SII registers of reimbursable medicines and Finnish outpatient registers.

### Statistical analyses

#### Latent class analysis to identify employment trajectories

Latent class analysis (LCA) was used to identify employment trajectories for combined role statuses at ages 16–45, using Mplus, version 8. Seven roles from the LHC were used to identify trajectories. Because gendered trajectories were expected [19], the analyses were performed separately by gender. In the LCA, the probability of occupying a specific status varies between zero and one. The estimation method was full information maximum likelihood method, and the link between latent categorical and observed dichotomous variables was logit.

To define the number of latent classes, we applied the adjusted Bayesian information criteria (aBIC), Lo–Mendell–Rubin likelihood ratio test (LMR-LRT) [20], average latent class posterior probabilities (AvePP) and entropy values that evaluated the discrimination among the latent classes. The AvePPs were calculated for the individuals with the highest posterior probability of being members of certain latent classes [21]. A value over 0.90 of the average value in the classes in which individuals showed the highest posterior probability described a clear class solution. Entropy was calculated using the average latent class probabilities, with values between zero and one. A high value indicated a high discriminant solution. Finally, to determine the number of latent classes, the clarity of the classes and the generalisability of the solution were evaluated by considering the classes’ descriptive in addition to the statistical criteria.

#### Characteristics of sample and trajectories

The background variables in the different diagnostic groups (SZ, OP and NP) were calculated separately by gender using cross-tabulation (categorical variables) and medians with interquartile ranges (continuous variables). Characteristics of men and women in the resulting employment trajectories (see below) were presented using cross-tabulation (categorical variables) and median with interquartile range (continuous variables).

#### Histories of psychoses and employment trajectories

The numbers of individuals with SZ or OP in various employment trajectories were compared to the numbers of NP in those trajectories.

Next, because both men and women with histories of psychosis were expected to cluster into the least favourable trajectories (later termed as floundering trajectories, see below), we continued comparing the distribution of pre-employment factors and illness onset ages in relation to belonging to a floundering employee trajectory versus other trajectories. The differences in such characteristics were evaluated using cross-tabulation (categorical variables) and medians with interquartile ranges (continuous variables).

Finally, we used logistic regression to examine the risk of experiencing the least favourable (floundering) employment trajectory in relation to a diagnosis of SZ or OP by using NP as a reference category. First, we conducted an unadjusted logistic regression. Then, we adjusted the regression separately for father’s SES at 14 years, average school grades at 16 years, educational level, marital status and SES at 46 years and lastly by fully adjusting for all these variables together. The results are presented as odds ratios (ORs) with 95% confidence intervals (CIs). The statistical analyses were conducted using IBM SPSS Statistics, version 25.

#### Attrition and weighted analysis

To account for the evident selected participation in relation to a history of psychosis in the 46 year survey and the formation of employment trajectories, we compared the participants’ educational levels, work situations and onset age of psychosis to those of non-participants using the register data. Based on this comparison, all analyses were repeated by using inverse probability weighting as a sensitivity analysis. See Online supplement 1 for further details.

#### Missing data

Regarding the sample’s characteristics, data on educational level were missing from 3 to 12% of people in different diagnostic categories; data on marital status from 0 to 5%; data on father’s SES from 13 to 21%; data on average school grades from 0 to 2%; data on socioeconomic status from 0 to 3%, and data on illness onset age from 0 to 26%. When analysing employment trajectories, data on father’s SES were missing from 13 to 33% of people; data on average school grades from 0 to 5%, and data on illness onset age, from 0 to 36%.

## Results

### Identification of employment trajectories

In identifying employment trajectories, the LMR-LRT suggested a three-class model for both men and women (Online supplement 2). The log-likelihood and aBIC continued decreasing for both genders from a one-class solution to a six-class solution. Entropy values remained high (over 0.95) for a five-class solution, as did the AvePPs. Summing up the statistical measures, men’s and women’s distinct characteristics in the five employment trajectories (Online supplement 3), in line with the solution in our previous study [12], a five-class solution was considered to best fit the data for both men and women, with meaningful profiles for the identified trajectories and with slight differences by gender.

**Fig. 1:**
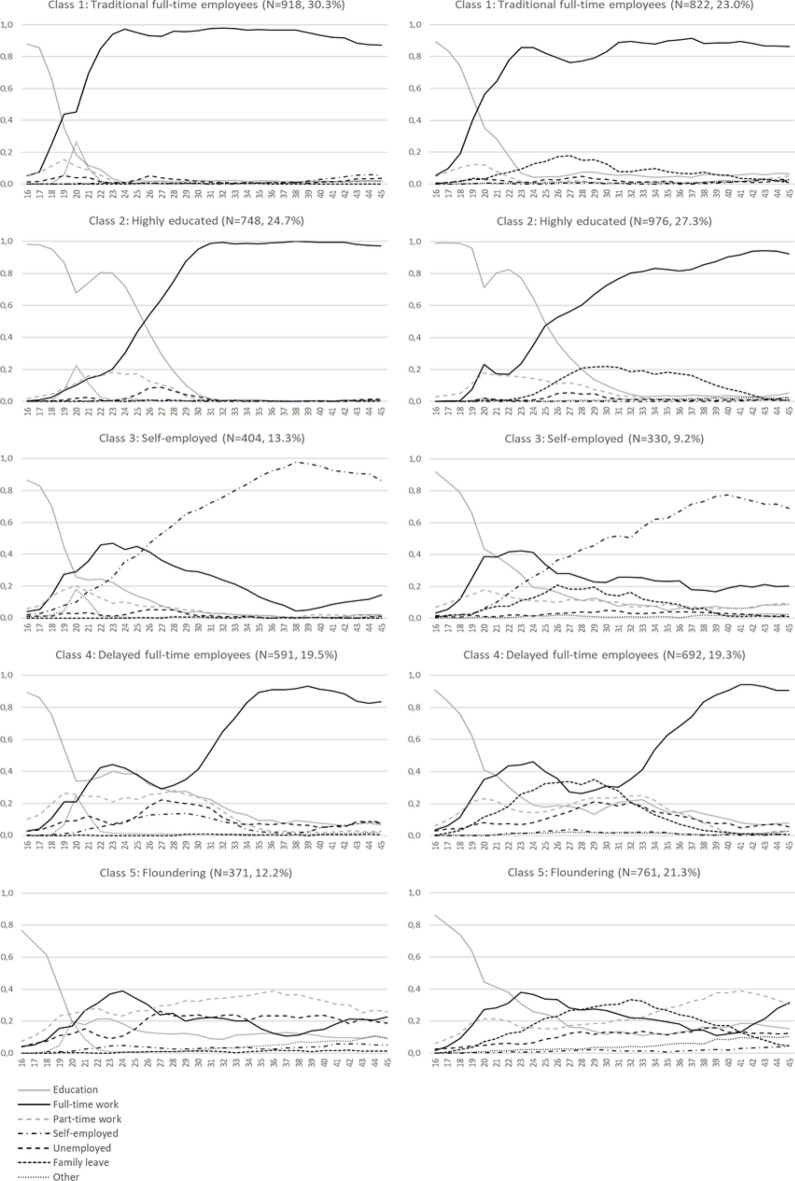
Five employment trajectories (probabilities of each employment role from 1982 to 2011, ages 16 to 45) found separately for men (left side) and for women (right side)

The yearly probabilities for each role status in each employment trajectory were calculated along with the total estimated proportion of membership for each trajectory (latent class; Fig. 1). The employment trajectories were named similarly to the previous study [12], as follows: (1) traditional full-time employees, (2) highly educated employees, 3) self-employed, (4) delayed full-time employees and (5) floundering employees.

The men and women traditional full-time employees’ trajectories were characterised by high probabilities of short education before progressing to full-time employment. The trajectories of highly educated employees were characterised by approximately 10 years of education before full-time employment. Individuals in self-employed trajectories were likely to have spent a few years in post-compulsory education and then some years in full-time employment before progressing to self-employment in early adulthood. Individuals in delayed full-time employees’ trajectories presented with elevated probabilities of part-time work, unemployment or parental leave during their early years in working life before entering full-time employment in their late 30 s. For individuals in floundering trajectories, part-time work and unemployment were common throughout the 30 year follow-up; in turn, full-time employment remained rare. In terms of education, in the floundering trajectories, the men were less educated than the women.

### Characteristics of the sample

Among persons with SZ, 47% were men, whereas 48% of those with OP and 46% of those with NP 46% were men. The individuals with SZ (50% of men, 79% of women) and OP (33% of men and women) were mainly on disability pensions. Secondary education was the most common educational level in the SZ group (75% of men, 87% of women), the OP group (57% of men, 67% of women) and the NP group (68% of men, 64% of women) (Table 1). In SZ, 14% of men and 10% of women, in OP 24% of men and 26% of women, and in NP 23% of men and 32% of women had attained tertiary education. The median age of psychosis onset was 29 years for men and 31 years for women with SZ, and 38 years for both men and women with OP (Table 1).

**Table 1:** Characteristics of the sample (*n* = 6613): *n* = 6464 with no psychosis, *n* = 87 with other psychosis, *n* = 62 with schizophrenia

Variable	Men			Women		
	No psychosis (*n* = 2961)	Other psychosis (*n* = 42)	Schizophrenia (*n* = 29)	No psychosis (*n* = 3503)	Other psychosis (*n* = 45)	Schizophrenia (*n* = 33)
Father’s SES at age 14, *n* (%)						
White collar	821 (33.0)	9 (27.3)	9 (37.5)	895 (30.0)	11 (28.2)	9 (33.3)
Other	1664 (67.0)	24 (72.7)	15 (62.5)	2089 (70.0)	28 (71.8)	18 (66.7)
Educational level by age 46, *n* (%)						
Basic or below	251 (8.9)	7 (18.9)	3 (10.7)	153 (4.6)	3 (7.0)	1 (3.2)
Secondary	1911 (67.8)	21 (56.8)	21 (75.0)	2105 (63.5)	29 (67.4)	27 (87.1)
Tertiary	655 (23.3)	9 (24.3)	4 (14.3)	1057 (31.9)	11 (25.6)	3 (9.7)
Marital status at age 46, *n* (%)						
Married/registered/cohabiting	2283 (79.1)	17 (42.5)	8 (27.6)	2688 (77.9)	23 (51.1)	11 (34.4)
Single/divorced/separated/widowed	602 (20.9)	23 (57.5)	21 (72.4)	762 (22.1)	22 (48.9)	21 (65.6)
Socioeconomic status at age 46, *n* (%)						
Farmer	92 (3.1)	2 (4.8)	1 (3.6)	51 (1.5)	1 (2.2)	0 (0.0)
Entrepreneur	322 (10.9)	1 (2.4)	0 (0.0)	213 (6.1)	1 (2.2)	0 (0.0)
Upper white collar	679 (23.0)	4 (9.5)	1 (3.6)	801 (22.9)	3 (6.7)	1 (3.0)
Lower white collar	608 (20.6)	4 (9.5)	2 (7.1)	1668 (47.7)	10 (22.2)	1 (3.0)
Manual worker	840 (28.4)	4 (9.5)	4 (14.3)	383 (11.0)	2 (4.4)	2 (6.1)
Student	37 (1.3)	0 (0.0)	2 (7.1)	65 (1.9)	1 (2.2)	0 (0.0)
Pensioner	61 (2.1)	14 (33.3)	14 (50.0)	69 (2.0)	15 (33.3)	26 (78.8)
Other	267 (9.0)	10 (23.8)	4 (14.3)	201 (5.8)	9 (20.0)	0 (0.0)
Unknown	47 (1.6)	3 (7.1)	0 (0.0)	43 (1.2)	3 (6.7)	3 (9.1)
Average school grades at age 16, Md (IQR)	7.3 (6.7–8.1)	7.0 (6.4–7.8)	7.4 (6.7–7.9)	8.1 (7.4–8.6)	7.9 (7.3–8.4)	7.7 (6.9–8.3)
Age at onset of psychosis, Md (IQR)		38.2 (31.1–42.0)	29.0 (24.0–38.4)		37.6 (33.3–42.9)	31.4 (25.7–34.2)

*SES* socioeconomic status*, Md* median, *IQR* interquartile range

### Employment trajectories in relation to psychoses

Most often, the individuals with SZ (79% of men, 73% of women) or OP (52% of men, 51% of women) had floundering trajectories (Table 2). Among those with NP, only 11% of men and 20% of women presented with floundering trajectories. In the NP group, men were most often (31%) in a traditional employee trajectory, whereas women were most likely to have a highly educated trajectory (28%).

**Table 2:** Distribution of employment trajectories between ages 16 and 45 in relation to a registered history of psychosis until age 46

	Men						Women					
	No psychosis (*n* = 2961)		Other psychosis (*n* = 42)		Schizophrenia (*n *= 29)		No psychosis (*n* = 3503)		Other psychosis (*n* = 45)		Schizophrenia (*n* = 33)	
	*n*	%	*n*	%	*n*	%	*n*	%	*n*	%	*n*	%
Employment trajectories												
Traditional employees	911	30.8	4	9.5	3	10.3	813	23.2	4	8.9	5	15.2
Highly educated employees	744	25.1	3	7.1	1	3.4	969	27.7	5	11.1	2	6.1
Self-employed	400	13.5	3	7.1	1	3.4	323	9.2	5	11.1	2	6.1
Delayed full-time employees	580	19.6	10	28.3	1	3.4	684	19.5	8	17.8	0	0.0
Floundering employees	326	11.0	22	52.4	23	79.3	714	20.4	23	51.1	24	72.7

Compared with the NP, a schizophrenia diagnosis was associated with a higher risk of having a floundering employee trajectory (OR (95% CI) 32.88 (13.28–81.39) for men, and 9.91 (4.56–21.50) for women). OP diagnoses were also associated with a risk of having a floundering employee category (OR (95% CI) 7.41 (3.96–13.85) for men and 3.88 (2.11–7.14) for women). After we fully adjusted for father’s SES at 14 years, average school grades at 16 years, educational level, marital status and SES at 46 years, the risk of belonging to the floundering employee category remained high in the SZ (OR (95% CI) 21.69 (7.43–63.34) for men and 6.48 (2.50–16.80) for women) and OP (OR (95% CI) 3.87 (1.70–8.82) for men and 3.35 (1.63–6.90) for women) groups. All ORs remained significant when adjusted separately for the same variables (Table 3).

**Table 3:** Odds ratios of belonging to the floundering employee trajectory vs. any other trajectories between ages 16 and 45 in relation to a history of psychosis by age 46

	Men			Women		
	OR (95% CI)			OR (95% CI)		
	No psychosis	Other psychosis	Schizophrenia	No psychosis	Other psychosis	Schizophrenia
Unadjusted	1	7.41 (3.96–13.85)	32.88 (13.28–81.39)	1	3.88 (2.11–7.14)	9.91 (4.56–21.50)
Adjusted						
Father’s SES at 14y	1	6.33 (3.11–12.88)	36.63 (13.38–100.25)	1	4.16 (2.17–7.98)	13.81 (5.55–34.37)
Average school grades at 16y	1	7.48 (3.93–14.24)	38.59 (15.30–97.30)	1	4.05 (2.17–7.53)	9.64 (4.43–21.00)
Educational level	1	6.19 (3.09–12.42)	33.20 (13.18–83.60)	1	3.81 (2.03–7.15)	8.79 (4.00–19.34)
Marital status	1	4.67 (2.38–9.16)	21.36 (8.37–54.48)	1	3.67 (1.99–6.78)	8.63 (3.93–18.91)
SES at 46y	1	5.98 (3.05–11.70)	25.44 (10.02–64.63)	1	3.00 (1.57–5.76)	5.13 (2.30–11.45)
Fully adjusted^a^	1	3.87 (1.70–8.82)	21.69 (7.43–63.34)	1	3.35 (1.63–6.90)	6.48 (2.50–16.80)

*OR* odds ratio, *CI* confidence interval, *SES* socioeconomic status

^a^Adjusted for father’s SES at 14y, average school grades at 16y, educational level, marital status, SES at 46y

The characteristics of men and women belonging to each employment trajectory are presented in (Table 4). In the SZ group, the median psychosis onset ages were 27 years for men and 30 years for women in the floundering trajectories and 41 for men and 32 for women in other trajectories. In the OP group, the corresponding ages were 35 for men and women in the floundering trajectories and 40 for men and 41 for women in the other trajectories.

**Table 4:** Pre-employment characteristics and illness onset age among men and women in floundering versus all other employment trajectories

Variable	No psychosis				Other psychosis				Schizophrenia			
	Men (*n* = 2961)		Women (*n* = 3503)		Men (*n* = 42)		Women (*n* = 45)		Men (*n* = 29)		Women (*n* = 33)	
	Floundering (*n* = 326)	Other (*n* = 2635)	Floundering (*n* = 714)	Other (*n* = 2789)	Floundering (*n* = 22)	Other (*n* = 20)	Floundering (*n* = 23)	Other (*n *= 22)	Floundering (*n* = 23)	Other (*n* = 6)	Floundering (*n* = 24)	Other (*n* = 9)
Father’s SES (14 y), *n* (%)												
White collar	57 (21.0)	764 (34.5)	177 (29.6)	718 (30.1)	3 (18.8)	6 (35.3)	6 (30.0)	5 (26.3)	8 (42.1)	1 (20.0)	7 (33.3)	2 (33.3)
Other	214 (79.0)	1450 (65.5)	421 (70.4)	1668 (69.9)	13 (81.3)	11 (64.7)	14 (70.0)	14 (73.7)	11 (57.9)	4 (80.0)	14 (66.7)	4 (66.7)
Average school grades at age 16, Md (IQR)	6.9 (6.4–7.6)	7.4 (6.8–8.1)	7.8 (7.2–8.5)	8.1 (7.4–8.7)	6.8 (6.4–7.2)	7.4 (6.7–8.5)	8.0 (7.7–8.3)	7.7 (7.2–8.4)	7.4 (6.6–7.8)	7.4 (7.0–8.0)	8.1 (7.4–8.4)	7.3 (6.7–7.5)
Age at onset of psychosis, Md (IQR)					34.6 (31.1–42.0)	39.5 (31.5–42.0)	35.4 (33.1–39.8)	40.7 (37.6–43.4)	27.3 (22.8–32.6)	41.4 (38.1–46.2)	30.4 (25.7–34.0)	31.6 (26.5–37.9)

*SES* socioeconomic status, *Md* median, *IQR* interquartile range

### Attrition analysis

Based on the registered data of the entire NFBC1966, 111/173 (64%) individuals with SZ, 87/174 (50%) with OP and 3,418/9,882 (35%) with NP did not participate in the current study. Among the cohort members with registered SZ diagnoses, men (*p* = 0.003) and women (*p* = 0.001) who did not participate in the 46 year survey tended to have lower levels of education than the participants. Non-participating men with SZ were less likely to be working than participating men with SZ (*p* = 0.028) (Online supplement 4). In the OP group, there were no statistically significant differences in any of the variables studied regarding participation. In the NP group, non-participants across genders had lower educational levels (*p* < 0.001 across genders) and were less likely to be working (*p* < 0.001 across genders) than the participants.

### Weighted analyses

The results of the weighted analyses were similar to those of the unweighted analyses (Online supplements 5–7).

## Discussion

### Main findings

To our knowledge, this is the first study to compare the distribution of typical employment trajectories in a general population birth cohort until midlife in relation to a lifelong history of SZ, OP or NP. We show that around three-quarters of men and women with SZ (73 and 79%, respectively) and about half of individuals with OP (51–52%) had floundering employment trajectories, characterised by continuously high probabilities of unemployment and part-time work compared to any other trajectory. The risk of having to a floundering employee trajectory versus other trajectories in midlife was very high for those with SZ (33-fold odds for men and tenfold odds for women) and OP (sevenfold odds for men and fourfold odds for women). These risks remained high even when adjusted for well-established potential confounders. The proportions of schizophrenic individuals in floundering employment trajectories are vastly greater than those of individuals with NP, whereas the corresponding distribution of people with OP is somewhere between these two groups.

### Comparison with previous studies

Our results align with previous studies which have reported that people with SZ are at high risk of being outside the labour market [2]. High proportions of individuals with SZ and OP in floundering employment trajectories indicate poor labour market attachment in these groups [22]. Our study supports previous findings on better occupational outcomes in people with OP than in SZ, but worse outcomes among these individuals than in people without psychosis [5, 23].

The rates of disability pension at age 46 in the SZ (50% in men, 79% in women) and OP (33% in men and women) groups in our sample were somewhat lower than the rates in the SZ (80–89%) [6, 7] and OP (69%) groups [7] in previous studies. This difference could be because individuals who receive disability pensions or have the most severe psychoses are less likely to participate in surveys, resulting in selection bias [24]. Our attrition analysis showed a high proportion (90%) of non-participating men with SZ who were not working and both men and women participants with SZ (as in the NP group) often having higher educational levels compared to non-participants. However, the results of the weighted analyses were highly similar to the non-weighted results.

Regarding education, our findings on low proportions of individuals with psychoses in highly educated trajectories align with a recent meta-analysis [25] demonstrating that compared to those without SZ, individuals with SZ attain significantly lower general academic achievement scores and are less likely to enter higher education. Interestingly, when comparing average school grades at age 16, men and women with subsequent floundering trajectories and who became diagnosed with SZ during the follow-up had school grades at least as high as those of men and women in the other trajectories, suggesting that success at school did not automatically lead to favourable employment. We also note that the age of illness onset was lower for men and women with SZ than for those with OP. In our sample, individuals with earlier onset ages were more likely to have floundering employee trajectories. Nevertheless, previous studies have presented higher cognitive functioning as a predictor of better vocational outcomes [26] and higher school grades as a predictor of a return to the labour market among individuals with SZ in the NFBC1966 [14].

The median age of psychosis onset in this study was high. This characteristic could be partly explained by the higher onset age among participants than non-participants as shown in the attrition analysis and by the higher number of people with OP compared to SZ among participants. Compared to SZ, age at onset of OP is generally higher [27], as in our sample. Moreover, the use of register data in defining the age of onset may affect, since the registers indicate the start of treatment instead of the start of actual symptoms. The association between earlier onset age and worse employment outcomes in SZ is unclear [26, 28], but some studies have found a significant relationship between these characteristics. However, later illness onset has been associated with many other beneficial outcomes in SZ [28].

Gender gaps in the labour market persist throughout the world [29]. The rates of floundering employees with SZ and those with OP in this study were similar across genders. Compared to men with SZ, women with SZ were more likely to be pensioners, whereas among participants with OP, the rate of pensioners was identical across genders. Compared to those with OP, individuals with SZ were more likely to be single and less likely to enter tertiary education. Previous studies have shown poorer outcomes in individuals with SZ than in those with OP [30] and poorer outcomes in men with SZ and OP than in women with SZ and OP [31]. Regarding occupational outcomes of schizophrenia, some studies have revealed better outcomes for females than for males [11, 32]. However, some studies have proposed better outcomes for males in terms of paid employment in some regions of the world [33].

### Clinical implications

The average time from the first psychiatric hospitalisation to disability pension in schizophrenia varies between 1 and 4 years [8, 34, 35]. A short time frame between illness onset and exit from the labour market in SZ and OP points to the importance of vocational rehabilitation and treatment at the early phases of such illnesses. Our results support the importance of early interventions that aim for a quick return to working life to prevent long-term exclusion.

Individuals with SZ often encounter barriers to gaining employment [3, 36]. Increasing access to rehabilitation services is associated with increased participation in employment [36]. However, receiving rehabilitative vocational treatment is insufficient to ensure labour stability, and there is a need for more comprehensive approaches that address the functional deficits that prevent people with SZ from obtaining employment [10]. A recent study of the 10 year employment trajectories of people with schizophrenia-spectrum disorders revealed that early intervention services may induce a long-term beneficial effect on employment [9]. An investment in first finding employment and thereafter tailoring care and rehabilitation—the Individual Placement and Support (IPS) approach—appears to increase employment rates for persons with psychosis, particularly during the intervention’s first months, and tends to be more effective than standard treatment [37]. A supported employment approach combined with interventions such as neurocognitive therapy and job-related skills training is useful because it is compatible with a wider range of employment outcomes and can be applied to many people [10]. However, more studies are needed on vocational rehabilitation and interventions that focus particularly on SZ and OP and differences in long-term employment trajectories.

It is important to acknowledge that in psychotic disorders, it is possible to return to the labour market after receiving a disability pension. Depending on the registers used, the length of follow-up and the type of disability pension, 9–19% of persons with psychotic disorders on disability pensions could return to the labour market [14]. It is essential to estimate whether current employment policies are effective in helping individuals with psychoses gain employment [23].

### Limitations and strengths

This study has some limitations. The sample had higher-level occupational and educational functioning than the general population, leading to selection bias. People with psychoses are often overrepresented in jobs that are relatively easy to perform [4, 38]. Thus, this study’s results may not be generalised to severely ill persons with psychoses. It should be noted, however, that the results remained similar in the weighted analyses. Our results are derived from a Nordic welfare country which provides access to education, social security and health care for all citizens, so the results are most generalisable to countries with corresponding labour market circumstances [39]. Because we conducted all the analyses separately by gender with respect to the gendered distribution of occupations, the resulting small sample sizes limit the results’ statistical power and possibilities for deep study of other than floundering trajectories and their potential predictors. Likewise, the small sample size limited the detailed examination of the subgroups of other psychotic diagnoses, such as psychotic depression. Moreover, some data were missing, which may have affected the results.

Nevertheless, our general population birth cohort sample from the NFBC1966 provides an important picture when comparing the distribution of employment trajectories of people with SZ and OP using data collected from registers and questionnaires. Our approach of focusing on the risk of experiencing the least favourable employment trajectories among those with psychoses is applicable across various societies. We managed to study 30 year-long working life trajectories from the ages of 16 to 45 years. One of the study’s strengths was the opportunity to study SZ and OP separately in the same sample instead of studying psychoses in general. In consideration of the differing distributions of occupations by gender, studying men and women separately allowed us to acknowledge the gender differences in longitudinal employment patterns in psychoses. Among men and women with floundering trajectories, the women had higher school grades and became more highly educated than men both in the overall sample and among those with psychoses. Another further strength of the study was the chance to minimise the effects of attrition and confounding factors using attrition analysis and adjusting for factors from childhood into adulthood.

## Conclusion

Schizophrenia and other psychoses present with a high risk for poor occupational outcomes with a longitudinally weak labour market attachment during working life until the age of 46. This study’s results can be utilised to improve labour market attachment in individuals suffering from psychotic illnesses and can be applied when planning vocational rehabilitation and interventions for these individuals. Our results support the importance of vocational rehabilitation starting during the early phases of psychotic illnesses.

## Supplementary Information

Below is the link to the electronic supplementary material.

Supplementary file1 (DOCX 20 KB)

Supplementary file2 (DOCX 15 KB)

Supplementary file3 (DOCX 21 KB)

Supplementary file4 (DOCX 21 KB)

Supplementary file5 (DOCX 20 KB)

Supplementary file6 (DOCX 16 KB)

Supplementary file7 (DOCX 16 KB)

## Data Availability

NFBC data are available from the University of Oulu, Infrastructure for Population Studies. Permission to use the data can be applied for research purposes via the electronic material request portal. Regarding the use of data, we follow the EU general data protection regulation (679/2016) and the Finnish Data Protection Act. The use of personal data is based on the cohort participant’s written informed consent at his/her latest follow-up study, which may cause limitations to its use. Please contact the NFBC Project Center (NFBCprojectcenter@oulu.fi) and visit the cohort website (www.oulu.fi/nfbc) for further information.
