# Supplementary material for: Employment trajectories until midlife in schizophrenia and other psychoses: the Northern Finland Birth Cohort 1966
Source: Soc Psychiatry Psychiatr Epidemiol. 2022 Jul 7;58(1):65–76. doi: 10.1007/s00127-022-02327-6 (PMC9845166; doi:10.1007/s00127-022-02327-6)
Supplement: Supplementary file 1 — Supplementary file1 (DOCX 20 KB) [file 127_2022_2327_MOESM1_ESM.docx]

Social Psychiatry and Psychiatric Epidemiology

Employment trajectories until midlife in schizophrenia and other psychoses – the Northern Finland Birth Cohort 1966

Tuomas Majuri^1^ · Anni-Emilia Alakokkare · Marianne Haapea · Tanja Nordström · Jouko Miettunen · Erika Jääskeläinen · Leena Ala-Mursula

^1^Center for Life Course Health Research, University of Oulu, Oulu, Finland.

Corresponding author:

BMed Tuomas Majuri,

email tuomas.majuri@student.oulu.fi

Online supplement 1

**Methods**

**Detecting individuals with a history of psychosis**

Psychiatric diagnoses of Northern Finland Birth Cohort 1966 (NFBC1966) members until the 46-year follow-up were retrieved from multiple national registers. The Care Register for Health Care (CRHC) [1] was used to find diagnoses of all general and psychiatric hospitalisations from 1974 and visits to specialised outpatient care since 1998. The Register of Primary Health Care Visits (2011–) [1] was used to find outpatient diagnoses in primary care. The data on lifetime diagnoses of individuals were complemented by register information from the Social Insurance Institution of Finland (SII) [2] on received special drug reimbursement (1974–2005), diagnoses for receiving sickness allowances (1974–1999), diagnoses for receiving disability pensions (1981–1998), and information from the Finnish Centre for Pensions (FCP) [3] on diagnoses for receiving disability pensions (1974–). The register data were complemented by self-reported lifetime-psychosis diagnosis, obtained by asking the participants in the 31- and 46-year questionnaire of the NFBC1966 whether they had ever been diagnosed by a physician as having psychosis. These people were assumed to have psychoses other than schizophrenia.

The focus was on individuals with schizophrenia (SZ) and other psychoses (OP) and for comparison purposes, the remaining cohort members (i.e., persons with no psychotic disorder (NP) in the national registers), were used as controls. When setting the diagnosis for each subject, we used a hierarchical system, in which the life-time diagnosis was the disorder that had the highest position in the hierarchy based on severity. The hierarchical order of diagnoses was from the most severe disorder to the least severe disorder in the following order: schizophrenia, other psychosis (including e.g., psychotic affective disorders and psychosis not otherwise specified) and no psychosis. For example, subjects diagnosed with SZ might have also had a diagnosis of some other psychotic disorder before or after schizophrenia, but their life-time diagnosis was interpreted as SZ. Subjects without psychotic disorders should not have had a diagnosis of psychosis in any of the registers or follow-up questionnaires because such a diagnosis would have moved them to the respective psychosis diagnostic group. This hierarchy has been used in previous studies of NFBC1966 [4].

1. Finnish Institute for Health and Welfare (2021) Register descriptions. https://thl.fi/en/web/thlfi-en/statistics-and-data/data-and-services/register-descriptions. Accessed 15 September 2021
2. The Social Insurance Institution of Finland (2021) Statistics. https://www.kela.fi/web/en/statistics. Accessed 15 September 2021
3. Finnish Centre for Pensions (2021) Statistics. https://www.etk.fi/en/research-statistics-and-projections/statistics/. Accessed 15 September 2021
4. Majuri T, Haapea M, Huovinen H et al (2021) Return to the labour market in schizophrenia and other psychoses: a register-based Northern Finland Birth Cohort 1966 study. Soc Psychiatry Psychiatr Epidemiol 56:1645-1655. https://doi.org/10.1007/s00127-020-02009-1

**Statistical analyses**

***Attrition and weighted analysis***

To account for an expected uneven participation in the 46-year survey and the formation of employment trajectories, we compared the characteristics (educational level, work situation and onset age of psychosis) of the participants of the 46-year follow-up questionnaire with information on employment trajectory available to non-participants using register data. Information on age of psychosis onset (until 2012), educational level (until 2015) and work situation (until 2012) was used. The data were complemented by information on educational level from the register of Statistics Finland and information on work situation in 2012 based on the registers of the Finnish Centre for Pensions (FCP) and the Social Insurance Institution of Finland (SII). For educational level, the same categories utilised when analysing the characteristics of the participants were used. The work situation was based on socio-economic status and was divided into two categories: 1) Working individuals, i.e. farmers, entrepreneurs, lower to upper white collar and manual workers and 2) Not working, i.e. students, pensioners and others. To observe the effect of attrition on the results, weighted analyses were conducted on the characteristics of the sample and the characteristics of the employment trajectories for both genders in the same diagnostic groups. The same variables used in the attrition analysis, i.e. educational level, work situation and age of illness onset (for psychoses only) in the same categories, were used as weights.

In the attrition analysis, the background variables in the different diagnostic categories (SZ, OP, NP) divided by gender were presented by participation using cross-tabulation and a Pearson’s chi-square test or Fisher’s exact test (categorical variables) and median with interquartile range and the Mann-Whitney test (continuous variables). P-values < 0.05 were considered statistically significant and all tests were two-tailed.

In the weighted analysis, characteristics of the sample and the trajectories of the different diagnostic categories (SZ, OP, NP) were weighted by inverse probability weighting and presented by gender using cross-tabulation (categorical variables) and median with interquartile range (continuous variables). The probability of belonging to the study group was calculated using logistic regression.

**Missing data in attrition analysis and weighted analyses**

In the attrition analysis, data on work situation were missing from 0–3% of males and 2–6% of females and data on illness onset age from 0–26% of males and 0–16% of females in different diagnostic groups divided by participation. Data of 58/62 with schizophrenia, 65/87 with other psychoses and 6357/6464 in non-psychotic group were available for the weighted analyses. In the weighted analyses, data on educational level were missing from 3–10%, data on father’s socioeconomic status from 15-30%, data on average school grades from 0–5%, data on illness onset age from 0–3% and data on marital status from 0–7% of people in different groups.
