# Supplementary material for: Employment trajectories until midlife in schizophrenia and other psychoses: the Northern Finland Birth Cohort 1966
Source: Soc Psychiatry Psychiatr Epidemiol. 2022 Jul 7;58(1):65–76. doi: 10.1007/s00127-022-02327-6 (PMC9845166; doi:10.1007/s00127-022-02327-6)
Supplement: Supplementary file 2 — Supplementary file2 (DOCX 15 KB) [file 127_2022_2327_MOESM2_ESM.docx]

Social Psychiatry and Psychiatric Epidemiology

Employment trajectories until midlife in schizophrenia and other psychoses – the Northern Finland Birth Cohort 1966

Tuomas Majuri^1^ · Anni-Emilia Alakokkare · Marianne Haapea · Tanja Nordström · Jouko Miettunen · Erika Jääskeläinen · Leena Ala-Mursula

^1^Center for Life Course Health Research, University of Oulu, Oulu, Finland.

Corresponding author:

BMed Tuomas Majuri,

email tuomas.majuri@student.oulu.fi

Online supplement 2

**Online supplement table 1.** Fit indices for the selection of the number of latent classes in yearly employment statuses (n=6,613)^a^

| Number of classes | Log-likelihood | aBIC | p-value of LMR-LRT | Entropy |
| --- | --- | --- | --- | --- |
| Males |  |  |  |  |
| 1 | -167992 | 336986 |  |  |
| 2 | -142654 | 287316 | <0.001 | 0.986 |
| 3 | -132698 | 268411 | <0.001 | 0.983 |
| 4 | -126343 | 256709 | 0.760 | 0.977 |
| 5 | -122992 | 251012 | 0.763 | 0.974 |
| 6 | -120169 | 246373 | 0.770 | 0.977 |
| Females |  |  |  |  |
| 1 | -232731 | 466514 |  |  |
| 2 | -213456 | 429020 | <0.001 | 0.968 |
| 3 | -206283 | 415730 | <0.001 | 0.977 |
| 4 | -201688 | 407597 | 0.762 | 0.954 |
| 5 | -198064 | 401403 | 0.763 | 0.953 |
| 6 | -195585 | 397503 | 0.773 | 0.953 |

*aBIC* adjusted Bayesian information criteria, *LMR-LRT* Lo-Mendell-Rubin likelihood ratio test

^a^For men, the AvePPs for the classes with the highest posterior probability in the five-class solution were 0.996, 0.989, 0.981, 0.994, and 0.985, and for women 0.959, 0.956, 0.994, 0.965, and 0.964.
