# Supplementary material for: Employment trajectories until midlife in schizophrenia and other psychoses: the Northern Finland Birth Cohort 1966
Source: Soc Psychiatry Psychiatr Epidemiol. 2022 Jul 7;58(1):65–76. doi: 10.1007/s00127-022-02327-6 (PMC9845166; doi:10.1007/s00127-022-02327-6)
Supplement: Supplementary file 3 — Supplementary file3 (DOCX 21 KB) [file 127_2022_2327_MOESM3_ESM.docx]

Social Psychiatry and Psychiatric Epidemiology

Employment trajectories until midlife in schizophrenia and other psychoses – the Northern Finland Birth Cohort 1966

Tuomas Majuri^1^ · Anni-Emilia Alakokkare · Marianne Haapea · Tanja Nordström · Jouko Miettunen · Erika Jääskeläinen · Leena Ala-Mursula

^1^Center for Life Course Health Research, University of Oulu, Oulu, Finland.

Corresponding author:

BMed Tuomas Majuri,

email tuomas.majuri@student.oulu.fi

Online supplement 3

**Online supplement table 2.** Characteristics of men and women in the five employment trajectories

|  | **Men (n=3032)** | | | | |  | **Women (n=3581)** | | | | |
| --- | --- | --- | --- | --- | --- | --- | --- | --- | --- | --- | --- |
|  | **Traditional full-time employees** | **Highly educated** | **Self-employed** | **Delayed full-time employees** | **Floundering** |  | **Traditional full-time employees** | **Highly educated** | **Self-employed** | **Delayed full-time employees** | **Floundering** |
| **Father’s SES at age 14, n (%)** |  |  |  |  |  |  |  |  |  |  |  |
| White collar | 199 (25.6) | 300 (47.0) | 116 (35.5) | 156 (31.6) | 68 (22.2) |  | 164 (23.5) | 342 (40.2) | 79 (29.3) | 140 (23.7) | 190 (29.7) |
| Other | 579 (74.4) | 338 (53.0) | 211 (64.5) | 337 (68.4) | 238 (77.8) |  | 535 (76.5) | 509 (59.8) | 191 (70.7) | 451 (76.3) | 449 (70.3) |
| **Educational level by age 46, n (%)** |  |  |  |  |  |  |  |  |  |  |  |
| Basic or below | 104 (11.8) | 2 (0.3) | 47 (12.1) | 37 (6.9) | 71 (20.5) |  | 50 (6.4) | 0 (0.0) | 25 (8.0) | 29 (4.4) | 53 (7.4) |
| Secondary | 723 (82.2) | 356 (49.6) | 297 (76.2) | 345 (63.0) | 232 (67.1) |  | 613 (78.5) | 402 (43.3) | 224 (72.0) | 440 (67.2) | 482 (67.5) |
| Tertiary | 53 (6.0) | 360 (50.1) | 46 (11.8) | 166 (30.3) | 43 (12.4) |  | 118 (15.1) | 526 (56.7) | 62 (19.9) | 186 (28.4) | 179 (25.1) |
| **Marital status at age 46, n (%)** |  |  |  |  |  |  |  |  |  |  |  |
| Married/registered/cohabiting | 687 (76.9) | 644 (87.6) | 336 (85.5) | 453 (78.8) | 188 (52.5) |  | 621 (77.0) | 765 (79.4) | 256 (78.5) | 539 (78.8) | 541 (72.3) |
| Single/divorced/separated/widowed | 206 (23.1) | 91 (12.4) | 57 (14.5) | 122 (21.2) | 170 (47.5) |  | 185 (23.0) | 198 (20.6) | 70 (21.5) | 145 (21.2) | 207 (27.7) |
| **Socioeconomic status at age 46,**  **n (%)** |  |  |  |  |  |  |  |  |  |  |  |
| Farmer | 0 (0.0) | 1 (0.1) | 87 (21.6) | 0 (0.0) | 7 (1.9) |  | 2 (0.2) | 1 (0.1) | 46 (14.0) | 0 (0.0) | 3 (0.4) |
| Entrepreneur | 48 (5.2) | 12 (1.6) | 202 (50.1) | 39 (6.6) | 22 (6.0) |  | 18 (2.2) | 19 (2.0) | 136 (41.5) | 9 (1.3) | 32 (4.2) |
| Upper white collar | 70 (7.6) | 409 (55.0) | 27 (6.7) | 155 (26.3) | 23 (6.2) |  | 86 (10.5) | 506 (52.1) | 15 (4.6) | 106 (15.3) | 92 (12.1) |
| Lower white collar | 222 (24.2) | 186 (25.0) | 26 (6.5) | 140 (23.7) | 40 (10.8) |  | 514 (62.6) | 384 (39.5) | 71 (21.6) | 414 (59.8) | 296 (39.0) |
| Manual worker | 472 (51.5) | 91 (12.2) | 41 (10.2) | 172 (29.2) | 72 (19.5) |  | 144 (17.5) | 32 (3.3) | 22 (6.7) | 96 (13.9) | 93 (12.3) |
| Student | 12 (1.3) | 4 (0.5) | 0 (0.0) | 9 (1.5) | 14 (3.8) |  | 9 (1.1) | 6 (0.6) | 10 (3.0) | 13 (1.9) | 28 (3.7) |
| Pensioner | 11 (1.2) | 1 (0.1) | 3 (0.7) | 2 (0.3) | 72 (19.5) |  | 10 (1.2) | 1 (0.1) | 12 (3.7) | 5 (0.7) | 82 (10.8) |
| Other | 74 (8.1) | 26 (3.5) | 13 (3.2) | 64 (10.8) | 104 (28.2) |  | 32 (3.9) | 18 (1.9) | 12 (3.7) | 43 (6.2) | 105 (13.8) |
| Unknown | 8 (0.9) | 14 (1.9) | 4 (1.0) | 9 (1.5) | 15 (4.1) |  | 6 (0.7) | 5 (0.5) | 4 (1.2) | 6 (0.9) | 28 (3.7) |
| **Average school grades at age 16, Md (IQR)** | 7.0 (6.5-7.5) | 8.0 (7.5-8.6) | 7.3 (6.8-7.9) | 7.4 (6.8-8.0) | 7.1 (6.5-7.6) |  | 7.7 (7.2-8.3) | 8.5 (8.1-8.9) | 7.9 (7.2-8.6) | 7.7 (7.2-8.4) | 7.81 (7.20-8.50) |

*SES* Socioeconomic status*, Md* median, *IQR* interquartile range
