# Supplementary material for: Employment trajectories until midlife in schizophrenia and other psychoses: the Northern Finland Birth Cohort 1966
Source: Soc Psychiatry Psychiatr Epidemiol. 2022 Jul 7;58(1):65–76. doi: 10.1007/s00127-022-02327-6 (PMC9845166; doi:10.1007/s00127-022-02327-6)
Supplement: Supplementary file 4 — Supplementary file4 (DOCX 21 KB) [file 127_2022_2327_MOESM4_ESM.docx]

Social Psychiatry and Psychiatric Epidemiology

Employment trajectories until midlife in schizophrenia and other psychoses – the Northern Finland Birth Cohort 1966

Tuomas Majuri^1^ · Anni-Emilia Alakokkare · Marianne Haapea · Tanja Nordström · Jouko Miettunen · Erika Jääskeläinen · Leena Ala-Mursula

^1^Center for Life Course Health Research, University of Oulu, Oulu, Finland.

Corresponding author:

BMed Tuomas Majuri,

email tuomas.majuri@student.oulu.fi

Online supplement 4

**Online supplement table 3.** Attrition analysis among men and women

| Variable |  | | |  | **Men** | | |  |  | | |
| --- | --- | --- | --- | --- | --- | --- | --- | --- | --- | --- | --- |
|  | **No psychosis** | | |  | **Other psychosis** | | |  | **Schizophrenia** | | |
|  | Participants (n=2961) | Non-participants (n=2057) | p-value |  | Participants (n=42) | Non-participants (n=46) | p-value |  | Participants (n=29) | Non-participants (n=73) | p-value |
| **Age at onset of psychosis, Md (IQR)** (until 2012) |  |  |  |  | 38.2  (31.3-42.0) | 36.9  (28.4-40.3) | 0.298 |  | 29.0  (24.6-38.1) | 29.7  (24.8-34.1) | 0.642 |
| **Educational level, n (%)** (until 2015) |  |  | <0.001 |  |  |  | 0.297 |  |  |  | 0.003 |
| Basic or below | 222 (7.5) | 342 (16.7) |  |  | 8 (19.0) | 9 (19.6) |  |  | 3 (10.3) | 25 (34.2) |  |
| Secondary | 1485 (50.2) | 1131 (55.0) |  |  | 22 (52.4) | 30 (65.2) |  |  | 17 (58.6) | 42 (57.5) |  |
| Tertiary | 1254 (42.4) | 584 (28.4) |  |  | 12 (28,6) | 7 (15.2) |  |  | 9 (31.0) | 6 (8.2) |  |
| **Work situation, n (%)** (2012) |  |  | <0.001 |  |  |  | 0.437 |  |  |  | 0.028^a^ |
| Working | 2541 (87.4) | 1543 (78.1) |  |  | 15 (38.5) | 14 (30.4) |  |  | 8 (28.6) | 7 (9.7) |  |
| Not working | 365 (12.6) | 433 (21.9) |  |  | 24 (61.5) | 32 (69.6) |  |  | 20 (71.4) | 65 (90.3) |  |

|  |  | | |  | **Women** | | |  |  | | |
| --- | --- | --- | --- | --- | --- | --- | --- | --- | --- | --- | --- |
|  | **No psychosis** | | |  | **Other psychosis** | | |  | **Schizophrenia** | | |
|  | Participants (n=3503) | Non-participants (n=1457) | p-value |  | Participants (n=45) | Non-participants (n=42) | p-value |  | Participants (n=33) | Non-participants (n=43) | p-value |
| **Age at onset of psychosis, Md (IQR)** (until 2012) |  |  |  |  | 37.6  (33.4-42.7) | 37.0  (30.4-41.9) | 0.253 |  | 31.4  (26.5-34.0) | 29.3  (21.9-34.8) | 0.268 |
| **Educational level, n (%)** (until 2015) |  |  | <0.001 |  |  |  | 0.092 |  |  |  | 0.001 |
| Basic or below | 122 (3.5) | 174 (11.9) |  |  | 5 (11.1) | 11 (26.2) |  |  | 0 (0.0) | 14 (32.6) |  |
| Secondary | 1376 (39.3) | 678 (46.5) |  |  | 20 (44.4) | 20 (47.6) |  |  | 22 (66.6) | 16 (37.2) |  |
| Tertiary | 2005 (57.2) | 605 (41.5) |  |  | 20 (44.4) | 11 (26.2) |  |  | 11 (33.3) | 13 (30.2) |  |
| **Work situation, n (%)** (2012) |  |  | <0.001 |  |  |  | 0.321 |  |  |  | 0.268 |
| Working | 3116 (90.3) | 1111 (79.4) |  |  | 17 (40.5) | 12 (30.0) |  |  | 4 (13.3) | 10 (23.8) |  |
| Not working | 335 (9.7) | 289 (20.6) |  |  | 25 (59.5) | 28 (70.0) |  |  | 26 (86.7) | 32 (76.2) |  |

*Md* median, *IQR* interquartile range

^a^P-value presented by Fisher’s exact test
