# Supplementary material for: Employment trajectories until midlife in schizophrenia and other psychoses: the Northern Finland Birth Cohort 1966
Source: Soc Psychiatry Psychiatr Epidemiol. 2022 Jul 7;58(1):65–76. doi: 10.1007/s00127-022-02327-6 (PMC9845166; doi:10.1007/s00127-022-02327-6)
Supplement: Supplementary file 5 — Supplementary file5 (DOCX 20 KB) [file 127_2022_2327_MOESM5_ESM.docx]

Social Psychiatry and Psychiatric Epidemiology

Employment trajectories until midlife in schizophrenia and other psychoses – the Northern Finland Birth Cohort 1966

Tuomas Majuri^1^ · Anni-Emilia Alakokkare · Marianne Haapea · Tanja Nordström · Jouko Miettunen · Erika Jääskeläinen · Leena Ala-Mursula

^1^Center for Life Course Health Research, University of Oulu, Oulu, Finland.

Corresponding author:

BMed Tuomas Majuri,

email tuomas.majuri@student.oulu.fi

Online supplement 5

**Online supplement table 4.** Characteristics of the weighted sample

|  | **Men** | | |  | **Women** | | |
| --- | --- | --- | --- | --- | --- | --- | --- |
| Variable | **No psychosis (n=2906)** | **Other psychosis (n=29)** | **Schizophrenia (n=28)** |  | **No psychosis (n=3451)** | **Other psychosis (n=36)** | **Schizophrenia (n=30)** |
| **Father’s SES at age 14, n (%)** |  |  |  |  |  |  |  |
| White collar | 772 (31.7) | 5 (26.2) | 7 (31.6) |  | 847 (29.0) | 7 (22.0) | 7 (35.4) |
| Other | 1660 (68.3) | 15 (73.8) | 15 (68.4) |  | 2078 (71.0) | 24 (78.0) | 13 (64.6) |
| **Educational level by age 46, n (%)** |  |  |  |  |  |  |  |
| Basic or below | 325 (11.8) | 6 (23.5) | 8 (28.8) |  | 202 (6.2) | 2 (5.6) | 1 (3.5) |
| Secondary | 1878 (68.0) | 15 (58.4) | 17 (63.9) |  | 2089 (64.1) | 27 (78.4) | 21 (74.87) |
| Tertiary | 558 (20.2) | 5 (18.0) | 2 (7.3) |  | 969 (29.7) | 6 (16.1) | 6 (21.7) |
| **Marital status at age 46, n (%)** |  |  |  |  |  |  |  |
| Married/registered/cohabiting | 2208 (78.1) | 13 (48.3) | 6 (19.8) |  | 2641 (77.8) | 16 (43.9) | 9 (30.6) |
| Single/divorced/separated/widowed | 620 (21.9) | 14 (51.7) | 22 (80.2) |  | 754 (22.2) | 20 (56.1) | 20 (69.4) |
| **Socioeconomic status at age 46, n (%)** |  |  |  |  |  |  |  |
| Farmer | 95 (3.3) | 2 (5.4) | 0 (1.4) |  | 53 (1.5) | 1 (3.3) | 0 (0.0) |
| Entrepreneur | 316 (10.9) | 1 (4.5) | 0 (0.0) |  | 211 (6.1) | 1 (2.2) | 0 (0.0) |
| Upper white collar | 595 (20.5) | 2 (7.3) | 0 (1.4) |  | 733 (21.2) | 2 (4.5) | 4 (13.9) |
| Lower white collar | 569 (19.6) | 2 (7.7) | 1 (2.7) |  | 1617 (46.9) | 7 (19.0) | 1 (3.8) |
| Manual worker | 858 (29.5) | 3 (10.7) | 2 (7.5) |  | 397 (11.5) | 3 (7.0) | 3 (9.1) |
| Student | 44 (1.5) | 0 (0.0) | 2 (5.6) |  | 78 (2.3) | 1 (2.8) | 0 (0.0) |
| Pensioner | 87 (3.0) | 10 (35.7) | 16 (55.6) |  | 102 (2.9) | 13 (37.1) | 22 (73.2) |
| Other | 342 (11.8) | 8 (28.6) | 7 (25.9) |  | 259 (7.5) | 9 (24.0) | 0 (0.0) |
| Unknown | 0 (0.0) | 0 (0.0) | 0 (0.0) |  | 0 (0.0) | 0 (0.0) | 0 (0.0) |
| **Average school grades at age 16, Md (IQR)** | 7.2 (6.6-8.0) | 6.8 (6.4-7.8) | 7.4 (6.9-7.7) |  | 7.9 (7.3-8.6) | 7.9 (6.9-8.3) | 7.7 (7.3-8.3) |
| **Age at onset of psychosis, Md (IQR)** |  | 36.1 (30.6-41.8) | 27.7 (21.4-38.6) |  |  | 37.4 (32.3-41.7) | 27.8 (20.5-33.1) |

*SES* Socioeconomic status*, Md* median, *IQR* interquartile range
