# Supplementary material for: Employment trajectories until midlife in schizophrenia and other psychoses: the Northern Finland Birth Cohort 1966
Source: Soc Psychiatry Psychiatr Epidemiol. 2022 Jul 7;58(1):65–76. doi: 10.1007/s00127-022-02327-6 (PMC9845166; doi:10.1007/s00127-022-02327-6)
Supplement: Supplementary file 6 — Supplementary file6 (DOCX 16 KB) [file 127_2022_2327_MOESM6_ESM.docx]

Social Psychiatry and Psychiatric Epidemiology

Employment trajectories until midlife in schizophrenia and other psychoses – the Northern Finland Birth Cohort 1966

Tuomas Majuri^1^ · Anni-Emilia Alakokkare · Marianne Haapea · Tanja Nordström · Jouko Miettunen · Erika Jääskeläinen · Leena Ala-Mursula

^1^Center for Life Course Health Research, University of Oulu, Oulu, Finland.

Corresponding author:

BMed Tuomas Majuri,

email tuomas.majuri@student.oulu.fi

Online supplement 6

**Online supplement table 5.** Distribution of the weighted employment trajectories in the study groups until 2012

|  | **Men** | | | | | |  | **Women** | | | | | |
| --- | --- | --- | --- | --- | --- | --- | --- | --- | --- | --- | --- | --- | --- |
|  | **No psychosis (n=2906)** | | **Other psychosis (n=29)** | | **Schizophrenia (n=28)** | |  | **No psychosis (n=3451)** | | **Other psychosis (n=36)** | | **Schizophrenia (n=30)** | |
|  | n | % | n | % | n | % |  | n | % | n | % | n | % |
| **Employment trajectories** |  |  |  |  |  |  |  |  |  |  |  |  |  |
| Traditional employees | 939 | 32.3 | 5 | 16.7 | 5 | 17.3 |  | 813 | 23.6 | 3 | 7.7 | 4 | 14.0 |
| Highly educated employees | 648 | 22.3 | 1 | 4.8 | 0 | 1.4 |  | 890 | 25.8 | 2 | 6.3 | 5 | 17.7 |
| Self-employed | 400 | 13.8 | 3 | 9.7 | 0 | 1.4 |  | 327 | 9.5 | 5 | 14.1 | 2 | 5.3 |
| Delayed full-time employees | 557 | 19.2 | 7 | 22.5 | 1 | 2.0 |  | 681 | 19.7 | 6 | 17.1 | 0 | 0.0 |
| Floundering employees | 362 | 12.5 | 13 | 46.3 | 22 | 77.9 |  | 739 | 21.4 | 20 | 54.8 | 19 | 63.0 |
