# Supplementary material for: Employment trajectories until midlife in schizophrenia and other psychoses: the Northern Finland Birth Cohort 1966
Source: Soc Psychiatry Psychiatr Epidemiol. 2022 Jul 7;58(1):65–76. doi: 10.1007/s00127-022-02327-6 (PMC9845166; doi:10.1007/s00127-022-02327-6)
Supplement: Supplementary file 7 — Supplementary file7 (DOCX 16 KB) [file 127_2022_2327_MOESM7_ESM.docx]

Social Psychiatry and Psychiatric Epidemiology

Employment trajectories until midlife in schizophrenia and other psychoses – the Northern Finland Birth Cohort 1966

Tuomas Majuri^1^ · Anni-Emilia Alakokkare · Marianne Haapea · Tanja Nordström · Jouko Miettunen · Erika Jääskeläinen · Leena Ala-Mursula

^1^Center for Life Course Health Research, University of Oulu, Oulu, Finland.

Corresponding author:

BMed Tuomas Majuri,

email tuomas.majuri@student.oulu.fi

Online supplement 7

**Online supplement table 6.** Characteristics of the weighted employment trajectories among men and women

| Variable | **No psychosis** | | | |  | **Other psychosis** | | | |  | **Schizophrenia** | | | | |
| --- | --- | --- | --- | --- | --- | --- | --- | --- | --- | --- | --- | --- | --- | --- | --- |
|  | **Men (n=2906)** | | **Women (n=3451)** | |  | **Men (n=29)** | | **Women (n=36)** | |  | **Men (n=28)** | | **Women (n=30)** | | |
|  | Floundering (n=361) | Other (n=2544) | Floundering (n=742) | Other (n=2710) |  | Floundering (n=13) | Other (n=16) | Floundering (n=21) | Other (n=16) |  | Floundering (n=22) | Other (n=6) | Floundering (n=19) | Other (n=11) |  |
| **Father’s SES (14 y), n (%)** |  |  |  |  |  |  |  |  |  |  |  |  |  |  |  |
| White collar | 64 (21.2) | 708 (33.2) | 171 (27.9) | 676 (29.2) |  | 1 (13.0) | 4 (34.6) | 4 (24.5) | 2 (18.7) |  | 7 (40.7) | 0 (6.5) | 6 (34.9) | 2 (37.1) |  |
| Other | 237 (78.8) | 1422 (66.8) | 441 (72.1) | 1637 (70.8) |  | 7 (87.0) | 8 (65.4) | 14 (75.5) | 11 (81.3) |  | 9 (59.3) | 5 (93.5) | 10 (65.1) | 3 (62.9) |  |
| **Average school grades at age 16, Md (IQR)** | 6.9 (6.4-7.5) | 7.3 (6.7-8.0) | 7.7 (7.1-8.4) | 8.0 (7.4-8.6) |  | 6.4 (6.3-7.3) | 7.1 (6.5-8.3) | 7.9 (6.9-8.3) | 7.7 (7.1-8.4) |  | 7.4 (6.6-7.9) | 7.0 (7.0-7.5) | 8.1 (7.4-8.4) | 7.6 (6.8-7.7) |  |
| **Age at onset of psychosis, Md (IQR)** |  |  |  |  |  | 33.9 (31.0-41.5) | 38.3 (29.2-42.1) | 35.0 (31.3-37.6) | 40.0 (32.1-41.9) |  | 25.7 (19.8-30.2) | 38.8 (38.7-43.5) | 28.5 (23.4-32.9) | 27.7 (19.8-37.2) |  |

*SES* Socioeconomic status, *Md* median, *IQR* interquartile range
